# Adenylyl Cyclase 6 Mediates Inhibition of TNF in the Inflammatory Reflex

**DOI:** 10.3389/fimmu.2018.02648

**Published:** 2018-11-27

**Authors:** Laura Tarnawski, Colin Reardon, April S. Caravaca, Mauricio Rosas-Ballina, Michael W. Tusche, Anna R. Drake, LaQueta K. Hudson, William M. Hanes, Jian Hua Li, William R. Parrish, Kaie Ojamaa, Yousef Al-Abed, Michael Faltys, Valentin A. Pavlov, Ulf Andersson, Sangeeta S. Chavan, Yaakov A. Levine, Tak W. Mak, Kevin J. Tracey, Peder S. Olofsson

**Affiliations:** ^1^Department of Medicine, Center for Bioelectronic Medicine, Center for Molecular Medicine, Solna, Karolinska Institutet, Karolinska University Hospital, Stockholm, Sweden; ^2^Department of Anatomy, Physiology & Cell Biology, University of California, Davis, Davis, CA, United States; ^3^Laboratory of Biomedical Science, The Feinstein Institute for Medical Research, Northwell Health, Manhasset, NY, United States; ^4^The Campbell Family Institute for Breast Cancer Research, University Health Network, Toronto, ON, Canada; ^5^SetPoint Medical, Valencia, CA, United States; ^6^Department of Biomedical Sciences, New York Institute of Technology College of Osteopathic Medicine, Old Westbury, NY, United States; ^7^Department of Women's and Children's Health, Karolinska Institutet, Stockholm, Sweden

**Keywords:** inflammatory reflex, choline acetyltransferase, acetylcholine, adenylyl cyclase 6, sustained TNF inhibition, vagus nerve stimulation/VNS, α7nAChR

## Abstract

Macrophage cytokine production is regulated by neural signals, for example in the inflammatory reflex. Signals in the vagus and splenic nerves are relayed by choline acetyltransferase^+^ T cells that release acetylcholine, the cognate ligand for alpha7 nicotinic acetylcholine subunit-containing receptors (α7nAChR), and suppress TNF release in macrophages. Here, we observed that electrical vagus nerve stimulation with a duration of 0.1–60 s significantly reduced systemic TNF release in experimental endotoxemia. This suppression of TNF was sustained for more than 24 h, but abolished in mice deficient in the α7nAChR subunit. Exposure of primary human macrophages and murine RAW 264.7 macrophage-like cells to selective ligands for α7nAChR for 1 h *in vitro* attenuated TNF production for up to 24 h in response to endotoxin. Pharmacological inhibition of adenylyl cyclase (AC) and knockdown of adenylyl cyclase 6 (AC6) or c-FOS abolished cholinergic suppression of endotoxin-induced TNF release. These findings indicate that action potentials in the inflammatory reflex trigger a change in macrophage behavior that requires AC and phosphorylation of the cAMP response element binding protein (CREB). These observations further our mechanistic understanding of neural regulation of inflammation and may have implications for development of bioelectronic medicine treatment of inflammatory diseases.

## Introduction

Infection and injury activate immune cells to produce cytokines and other factors that mediate inflammation. Macrophage function is critical for protection from infection and injury. Animals rendered deficient in macrophages, or subjected to genetic modifications that prevent cytokine production, have a diminished inflammatory response. During inflammation, the cellular response can be sustained for hours or days. Recent discoveries reveal that the responsiveness of innate immune cells when re-encountering pathogens is modulated by transcription factors and epigenetic reprogramming (1). However, the mechanisms initiating these changes that regulate inflammation are not fully understood.

Glucocorticoids, anti-inflammatory cytokines, immunoresolvents, and other humoral anti-inflammatory agents regulate immune responses and the resolution of inflammation, e.g. by inhibiting macrophage production of pro-inflammatory cytokines (2). Neural reflex circuits also regulate macrophage release of pro-inflammatory cytokines (3–6). Electrical activation of the vagus nerve (milliseconds to minutes) can significantly reduce endotoxin-induced release of Tumor Necrosis Factor (TNF), IL-1, IL-6, and HMGB1 (7, 8). Data from experimental disease models and clinical studies using vagus nerve stimulation (VNS) to treat rheumatoid arthritis and Crohn's disease indicate that transient electrical stimulation of the vagus nerve results in a prolonged alleviation of inflammation and disease severity (9–12). However, the mechanism underlying this sustained reduction of cytokine production in response to transient nerve activation has been unknown.

It was previously established that action potentials in the vagus nerve culminate in activation of the α7 nicotinic acetyl choline receptor subunit (α7nAChR) on macrophages (4, 13–15). The molecular signal that completes this inhibitory reflex requires an intact splenic nerve, and choline acetyltransferase^+^ (ChAT^+^) T cells that release acetylcholine (ACh), the cognate ligand for the α7nAChR (5). Several mechanisms by which nAChRs control inflammatory responses have been proposed, including activation of JAK2/STAT3 and inhibition of NFκB (16–19). The α7nAChR is an ion channel in neurons (20) but reportedly, the ion flux itself is not sufficient to mediate inhibition of TNF release (21), suggesting involvement of other molecular components.

In neurons, signal transduction via α7nAChR increases cyclic adenosine monophosphate (cAMP) levels and phosphorylation of the transcription factor cAMP response element binding protein (CREB) (22), a pathway that is essential for memory (23). In macrophages, increased cAMP promotes intracellular accumulation of the transcription factor c-FOS, and promotes suppression of inflammatory cytokine production (24). Interestingly, CREB has been implicated in cAMP-induced inhibition of NFκB-mediated transcription of TNF (25). Accordingly, we hypothesized that a cAMP and CREB-mediated increase in *c-Fos* expression underlies the development of persistently attenuated macrophage responses after VNS.

Here, we observed that VNS ≥24 h prior to an inflammatory insult reduced endotoxin induced TNF release through a mechanism that required the α7nAChR. Cholinergic inhibition of TNF in macrophages was abolished by inhibition of adenylyl cyclase, CREB phosphorylation, and expression of *c-Fos*.

## Results

### Vagus nerve stimulation inhibits cytokine release for more than 24 h

**Figure 1:**
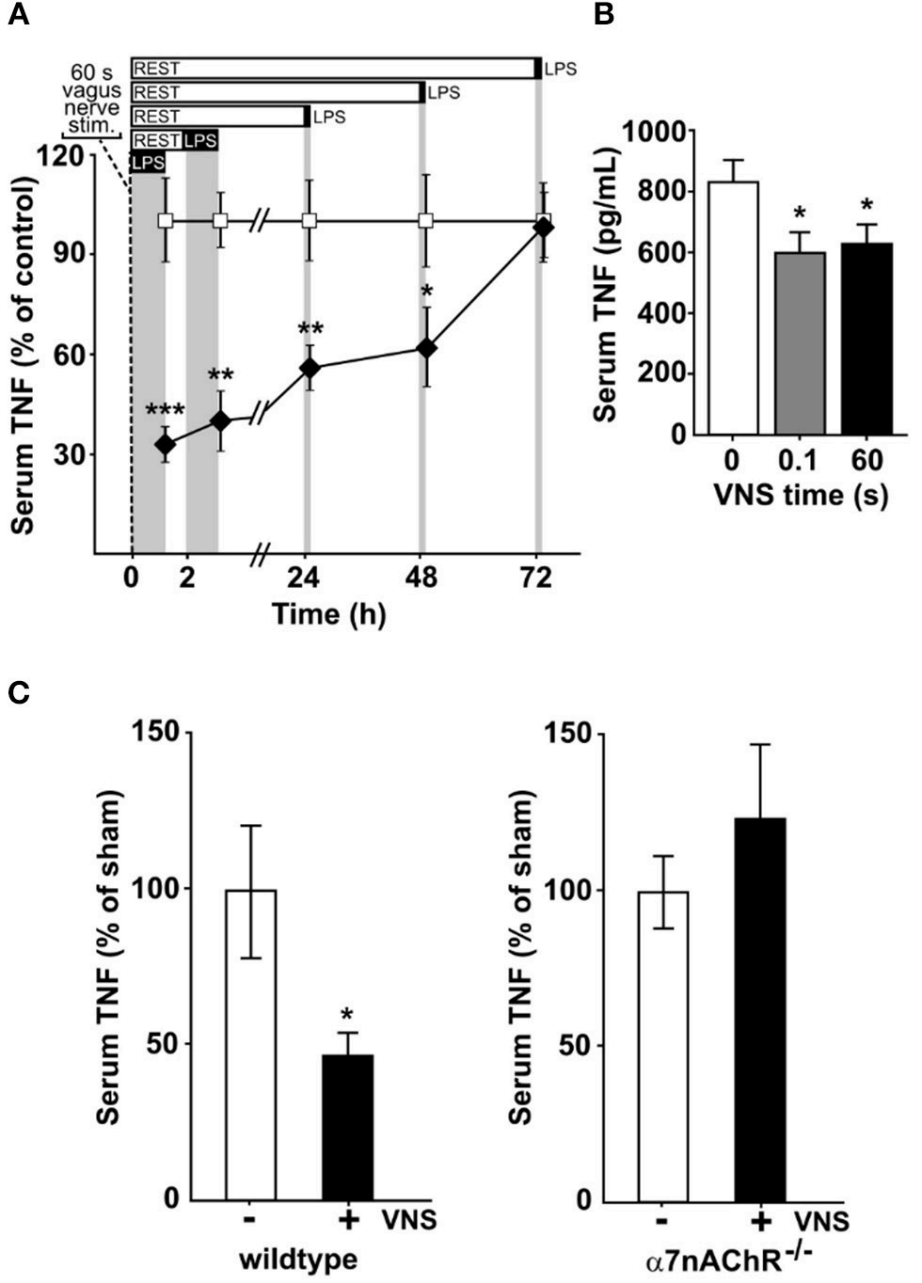
Vagus nerve stimulation suppresses endotoxin-induced serum TNF levels for days. Animals were subjected to vagus nerve stimulation (VNS) at 10 Hz followed by intraperitoneal endotoxin (LPS) injection at a specified time after VNS and then euthanized 90 min after endotoxin administration. Serum was collected and analyzed for TNF by ELISA. **(A)** Rats (*n* = 4–13/group) were subjected to 60 s of VNS or sham surgery. Open squares: mean TNF ± SEM in sham animals, filled diamonds: mean TNF ± SEM in vagus nerve stimulated animals. **(B)** Mice were subjected to 0 (*n* = 9), 0.1 s (*n* = 7), or 60 s (*n* = 9) of VNS and rested for 24 h before endotoxin injection. Means ± SEM are plotted. **(C)** Wild type (*n* = 7; left) and α7nAChR^−/−^ (*n* = 7; right) male mice were subjected to 60 s of VNS or sham surgery and rested for 24 h before endotoxin injection. TNF levels relative to unstimulated animals are shown as mean ± SEM. ^***^p < 0.001, ^**^p < 0.01, ^*^p < 0.05.

To study the kinetics of TNF release in endotoxemia following VNS, we subjected rats to vagus nerve stimulation (VNS) for 60 s at 10 Hz, or sham surgery. The rats were then injected intraperitoneally with bacterial endotoxin (lipopolysaccharide, LPS) at various time points after VNS. Serum TNF levels were significantly lower in VNS-treated rats compared to sham treated animals when endotoxin was administered 0, 2, 24, and 48 h after stimulation (Figure 1A). The VNS-mediated inhibition of endotoxin-induced TNF was not significant at 72 h after nerve stimulation.

Next, mice were treated with 60 s of VNS at 10 Hz, or sham surgery, and subjected to endotoxemia 24 h after treatment. Endotoxin-induced TNF levels were significantly lower in VNS- treated mice as compared to sham treated animals (Figure 1B). We previously observed that a single pulse, equivalent to 0.1 s stimulation, is sufficient to significantly reduce TNF release in experimental endotoxemia (8). To investigate if a single pulse confers a sustained reduction in TNF release, mice treated with 0.1 s of VNS were subjected to endotoxemia, 24 h after treatment. Endotoxin-induced TNF levels were significantly lower in VNS treated mice compared to sham treated animals (Figure 1B).

Together, these results suggest that a short activation of the inflammatory reflex reduces TNF production during endotoxemia for ≥24 h.

### α7nACHR is required for the sustained inhibition of cytokine release *in vivo*

Prior work established that the α7 nicotinic acetylcholine receptor subunit (α7nAChR) expressed by macrophages is required for the functional integrity of the inflammatory reflex (14, 15, 26). To study the role of α7nAChR in the persistent inhibition by VNS of TNF release in endotoxemia, we next studied α7nAChR deficient and wild type mice. Endotoxin-induced TNF was significantly reduced at 24 h after VNS in wild type mice (Figure 1C, left panel), but not in α7nAChR deficient mice (Figure 1C, right panel). Thus, α7nAChR is required for the persistent inhibition of TNF release by transient electrical stimulation of the vagus nerve.

### Exposure of macrophages to selective α7nACHR agonists increases intracellular cAMP levels

α7nAChR subunits form ion channels on neuronal cells (20). Although ion flux has been observed in macrophages exposed to cholinergic agonists, this effect is reportedly unlikely to affect cytokine production (21).

**Figure 2:**
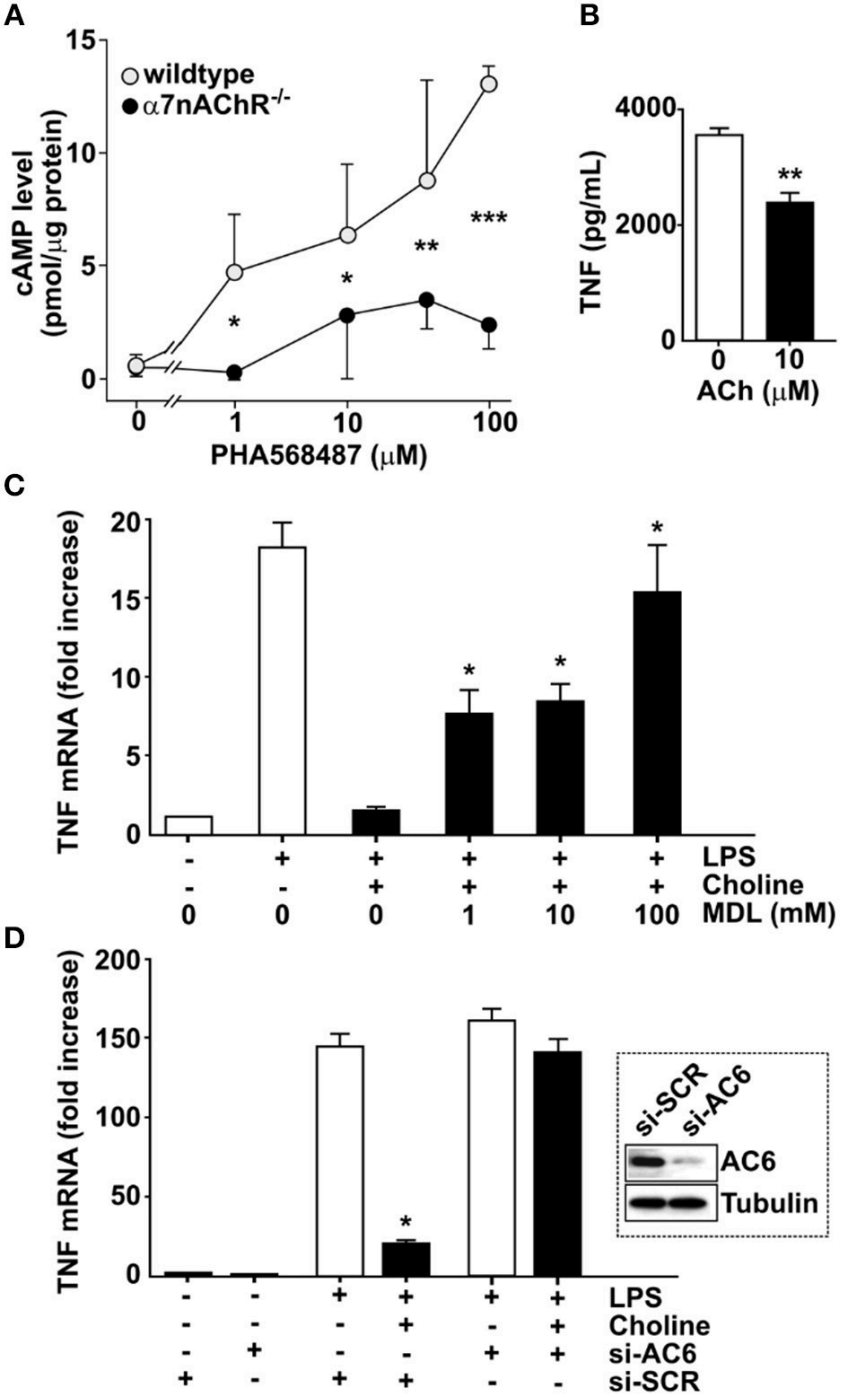
Adenylyl cyclase 6 mediates inhibition of TNF. **(A)** Bone-marrow derived macrophages from wild type (*n* = 2) and α7nAChR ^−/−^ (*n* = 3) mice were exposed to the α7nAChR selective agonist PHA568487 and cAMP analyzed in the cell lysate. Results are shown as mean cAMP levels normalized to protein concentration (pmol/μg) ± SEM. Open circles—wild type, filled circles—α7nAChR ^−/−^ cells. **(B)** RAW 264.7 macrophage-like cells were incubated with 10 μM acetylcholine for 1 h. The ACh was removed and 24 h post ACh, the cells were exposed to endotoxin (LPS) for 4 h. TNF was measured in the culture medium using ELISA. Plotted values are mean ± SEM. **(C)** RAW 264.7 cells were incubated with the adenylyl cyclase inhibitor MDL 12,330A, then exposed to choline and endotoxin. Values shown are mean fold increase of endotoxin-induced TNF mRNA ± SEM relative to cells exposed to endotoxin and choline in the absence of MDL 12,330A. **(D)** Adenylyl cyclase 6 (AC6) was knocked down using siRNA in RAW 264.7 cells which were then exposed to the α7nAChR selective agonist choline. Western blot shows AC6 in cells treated with siRNA targeting AC6 (si-AC6) or scrambled siRNA (si-SCR). Bars represent fold increase ± SEM of TNF mRNA compared to cells not challenged with endotoxin. ^*^p < 0.05, ^**^p < 0.01, ^***^p < 0.001.

In neurons, α7nAChR can interact with adenylyl cyclase 6 (AC6) and promote cAMP formation (27, 28). To investigate the possibility that a similar interaction occurs in macrophages, bone marrow derived macrophages from α7nAChR ^+/+^ and α7nAChR ^−/−^ mice, respectively, were exposed *in vitro* to the selective α7nAChR agonist, *N*-(3*R*)-1-Azabicyclo[2.2.2]oct - 3-yl - 2,3 - dihydro - 1,4-benzodioxin - 6 - carboxamide fumarate (PHA568487) (29). Addition of PHA568487 significantly increased cAMP levels in α7nAChR ^+/+^, but not in α7nAChR ^−/−^ derived macrophages (Figure 2A), indicating that that α7nAChR signaling regulates cAMP in murine macrophages.

To study the mechanism of cholinergic control of TNF release we utilized macrophage-like RAW 264.7 cells. First, we investigated whether cholinergic activation confers prolonged inhibition of endotoxin-induced TNF release in RAW 264.7 cells. Cells were exposed to 10 μM ACh or vehicle for 1 h, and then washed and re-suspended in fresh medium. Cells were then rested for 24 h and subsequently exposed to endotoxin for 4 h. TNF levels in culture medium were significantly lower in cultures treated with ACh (Figure 2B) compared to cultures treated with vehicle. To investigate whether blocking of cAMP synthesis prevents cholinergic inhibition of endotoxin-induced TNF expression, RAW 264.7 cells were cultured in the presence of the adenylyl cyclase-inhibitor [cis-N-(2-Phenylcyclopentyl)azacyclotridec-1-en-2-amine.HCl] (MDL 12,330A) and exposed to the selective α7nAChR agonist choline for 30 min. The cells were then incubated with endotoxin for 3 h and TNF mRNA was measured in cell lysates by qPCR. Choline-mediated suppression of TNF mRNA was significantly reduced in the presence of MDL 12,330A (Figure 2C). Based on the observations that α7nAChR can interact with AC6 (27) and that blocking cAMP synthesis reduced cholinergic inhibition of endotoxin-induced TNF expression, RAW 264.7 cells were transfected with siRNA targeting AC6 or scrambled siRNA. AC6 protein knock-down was confirmed by Western blot (Figure 2D, inset). Transfected cells were subsequently incubated with choline and cultured in the presence of endotoxin for 3 h. Knock down of AC6 cells abolished the choline-dependent reduction of TNF mRNA (Figure 2D), indicating that AC6 is required for the α7nAChR-mediated reduction of endotoxin-induced TNF mRNA. Together, these data indicate that cholinergic inhibition of endotoxin-induced TNF expression requires AC6-dependent cAMP production.

### *c-Fos* is essential for α7nACHR-mediated inhibition of TNF

**Figure 3:**
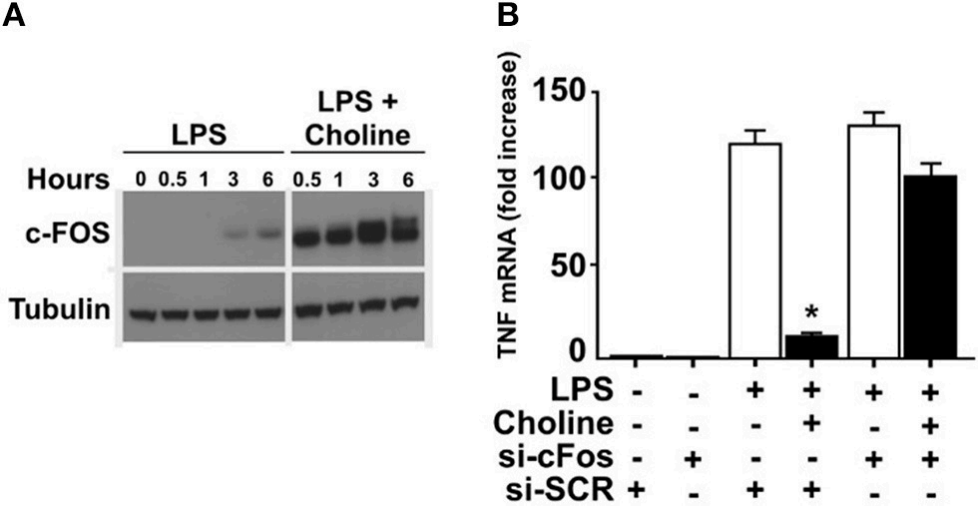
c-FOS mediates inhibition of TNF. **(A)** RAW 264.7 macrophage-like cells were exposed to endotoxin (LPS) only, or endotoxin together with the α7nAChR selective agonist choline. C-FOS was measured at 0 −6 h after endotoxin exposure by Western blot. **(B)** *c-Fos* was knocked down using siRNA in RAW 264.7 cells which were subsequently exposed to choline. Endotoxin-induced TNF mRNA levels were measured by qPCR. Bars represent fold increase ± SEM of TNF mRNA compared to cells not challenged with endotoxin. Data is representative of three independent experiments. ^*^p < 0.05.

It was previously established that increased cAMP levels promote expression and phosphorylation of c-FOS, which regulates macrophage TNF expression (24). Accordingly, we measured c-FOS protein in endotoxin-exposed RAW 264.7 cells in the presence of choline or vehicle (Figure 3A). Addition of choline augmented the production of c-FOS, supporting involvement of c-FOS in α7nAChR-mediated regulation of TNF. Based on these observations, we hypothesized that increased *c-Fos* expression in response to selective α7nAChR agonists contributes to the cholinergic inhibition of TNF production in macrophages. To test this, RAW 264.7 cells were transfected with siRNA targeting *c-Fos* or scrambled siRNA. Transfected cells were subsequently incubated with choline and cultured in the presence of endotoxin for 3 h. In cells transfected with scrambled siRNA, choline significantly inhibited endotoxin-induced TNF. In contrast, the inhibitory effect on endotoxin-mediated TNF expression was completely abolished in cells transfected with siRNA targeting *c-Fos* (Figure 3B). These data demonstrate that *c-Fos* is required for cholinergic suppression of endotoxin-induced TNF expression.

### Phosphorylation defective CREB prevents α7nACHR-mediated inhibition of TNF

**Figure 4:**
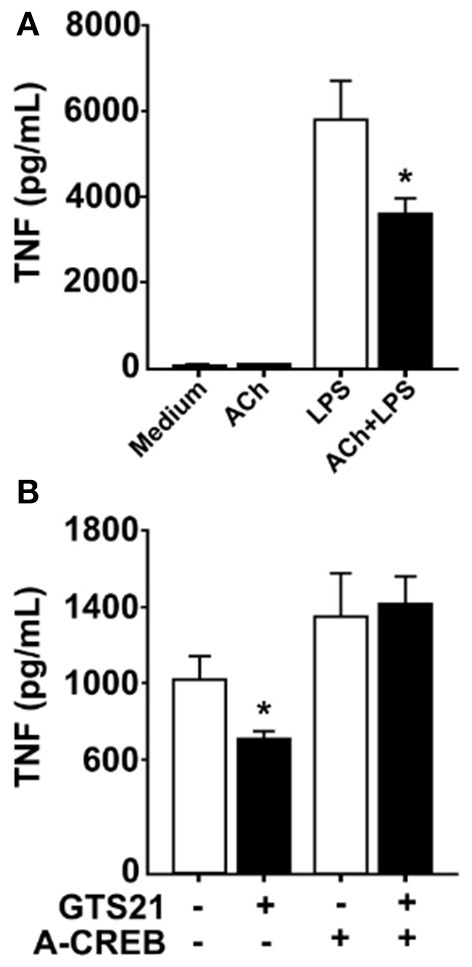
Functional CREB mediates persistent inhibition of TNF. **(A)** Human macrophages were incubated *in vitro* with acetylcholine (ACh), washed and rested for 24 h, and subsequently exposed to endotoxin (LPS) for 4 h. TNF was measured in the culture media using ELISA. **(B)** Primary human macrophages were transduced with adenovirus engineered to encode a dominant negative phosphorylation defective CREB (ACREB), or a GFP-expressing control adenovirus. After viral transduction, cells were stimulated with the selective α7nAChR agonist GTS21, washed, and cultured for 24 h before exposure to endotoxin. TNF was measured in culture media by ELISA. Mean TNF levels ± SEM in cells transduced with control virus or the phosphorylation defective ACREB-expressing virus (ACREB) are shown. ^*^p < 0.05.

In neurons, signal transduction via α7nAChR leads to increased cAMP levels and phosphorylation of the transcription factor CREB (22), a pathway that is essential for long-term retention of information, i.e., memory (23). CREB has also been implicated in cAMP-induced inhibition of NFκB-mediated transcription of TNF (25) and activation of CREB is known to promote expression of *c-Fos* (30, 31). RAW 264.7 cells and human macrophages cultured in the presence of ACh or vehicle showed increased CREB phosphorylation in cultures exposed to ACh as measured by western blot (data not shown). Accordingly, we hypothesized that CREB phosphorylation underlies sustained cholinergic reduction of endotoxin-induced TNF release in macrophages. To study this, we first established whether cholinergic stimulation of human differentiated macrophages confers sustained inhibition of TNF release. Human macrophages *in vitro* were exposed to ACh or vehicle for 1 h, the ACh washed away, and the cells rested for 24 h. Subsequently, cultures were exposed to endotoxin for 4 h and TNF levels were measured in culture medium. ACh exposure significantly reduced endotoxin-induced TNF release to the medium 24 h after ACh exposure (Figure 4A), demonstrating that ACh exposure confers persistent reduction in endotoxin-induced TNF release in human macrophages. Next, human macrophages were transduced with a phosphorylation defective CREB (ACREB), using an engineered adenovirus (32). TNF production by human primary macrophages transduced with control virus was significantly inhibited at 24 h after exposure to the selective α7nAChR agonist 3-(2,4-dimethoxybenzylidene)anabaseine (GTS21; Figure 4B). However, GTS21 failed to elicit prolonged deactivation of macrophages transduced with the ACREB containing adenovirus (Figure 4B), indicating that CREB phosphorylation is required for the sustained inhibition of cytokine release.

## Discussion

The results here indicate that electrical stimulation of the vagus nerve for 0.1–60 s reduces endotoxin-induced TNF release for ≥24 h by a mechanism that requires the α7nAChR subunit. Exposure of macrophages to selective α7nAChR agonists for 1 h resulted in prolonged inhibition of endotoxin-induced TNF release. The effect required adenylyl cyclase, CREB phosphorylation and c-FOS. Thus, we observe that macrophages retain information entrained by electrical signals in the vagus nerve.

VNS pulse trains shorter than 10 min significantly reduces inflammation and release of pro-inflammatory cytokines in experimental models of inflammation and clinical trials (9, 11, 12, 15). In the present study, one minute of VNS attenuated the systemic response to endotoxin for ≥24 h in mice and rats *in vivo*. For the acute effect of VNS on endotoxin-induced TNF release, the α7nAChR subunit is required (14, 15). The prolonged inhibition of endotoxin-induced TNF release observed here also required the α7nAChR subunit, as VNS failed to reduce endotoxin-induced TNF release 24 h after stimulation in α7nAChR^−/−^ mice. Priming of macrophages with a cholinergic agonist resulted in significant attenuation of TNF production 24 h after agonist removal, revealing a mechanism that can persist for hours after a transient nerve signal.

Cholinergic ligands are known to reduce NFκB activation and TNF release in macrophages (18, 19). de Jonge and colleagues concluded that nicotine increases phosphorylation of STAT3 and reduced TNF release from peritoneal macrophages without affecting TNF transcription (16). Others have shown that increased cAMP levels attenuate TNF expression through upregulation of *c-Fos* and direct inhibition of NFκB binding to the TNF promoter (24, 33). The cholinergic inhibition of TNF mRNA expression in the present study was abrogated by the adenylyl cyclase inhibitor MDL 12,330A. A mechanistic role for cAMP in this context was further supported by our observation that knock-down of AC6 and c-FOS, respectively, abolished cholinergic attenuation of TNF transcription. Accordingly, these results indicate that cholinergic inhibition of TNF transcription and perhaps also release likely involves cAMP- and *c-Fos*-mediated transcriptional changes.

**Figure 5:**
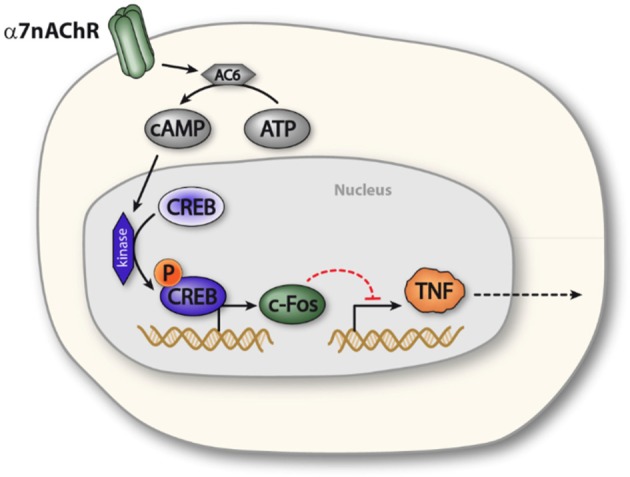
Proposed model of α7nAChR mediated prolonged regulation of TNF in macrophages. Activation of α7nAChR promotes activity of adenylyl cyclase 6, which increases production of cAMP. Increased intracellular levels of cAMP promotes phosphorylation of CREB, which enhances expression of c-FOS, c-FOS, in turn, is involved in NFκB mediated inhibition of TNF transcription. In this way, α7nAChR activation can inhibit endotoxin-induced TNF production.

The α7nAChR subunit has been implicated in long term neuronal memory formation (23), a process in which the transcription factor CREB also plays a key role (22). CREB was furthermore reported to modulate cytokine production in macrophage-like cells (34) and has been implicated in cAMP-mediated regulation of *c-Fos* expression (25). In the present study, functional inactivation of CREB abolished the cholinergic inhibition of TNF release in macrophages. Based on this observation, we propose the possibility that α7nAChR activation elicits CREB-dependent transcriptional changes in macrophages, which reduces endotoxin-induced TNF release (Figure 5).

Taken together, the results here identify mechanistic elements involved in the sustained reduction of TNF release after electrical vagus nerve stimulation and show that the cholinergic neurotransmitter acetylcholine can potentially confer a prolonged change in macrophage activation behavior. The resulting inhibition of TNF may attenuate excessive systemic inflammation.

In conclusion, our findings indicate that macrophages retain information from cholinergic signals. This mechanism requires the α7nAChR subunit, AC6, cAMP synthesis, phosphorylation of CREB, and expression of c-Fos. This data improves our understanding of the inflammatory reflex and potentially opens new avenues for therapeutic advancements in treatment of excessive inflammation.

## Materials and methods

### Ethical statement

Experimental *in vivo* protocols were approved by the Institutional Animal Care and Use Committee (IACUC) at the Feinstein Institute for Medical Research, Northwell Health, which follows the National Institutes of Health guidelines for the ethical treatment of animals. Animals were housed under a standard 12 h light/dark cycle and acclimatized for at least 1 week before conducting experiments. Water and regular rodent chow were available *ad libitum*. Human PBMCs from blood donors derive from completely anonymized, excess leukocytes, which do not require ethical approval.

### Vagus nerve stimulation

Male Sprague-Dawley rats were anesthetized using ketamine (100 mg/kg) and xylazine (10 mg/kg; IP). The left carotid sheath containing the vagus nerve was isolated and stimulated for 60 s at 1.0 mA, 10 Hz frequency, 0.25 ms biphasic pulse using a bipolar electrode (MicroProbes for Life Science). In sham-operated animals, the vagus nerve was visualized but not manipulated.

Male C57Bl/6, wild type and α7nAChR deficient, mice were anesthetized with ketamine (100 mg/kg) and xylazine (10 mg/kg), IP. A midline cervical incision was made, the salivary glands separated, and the left carotid sheath containing the vagus nerve was isolated between the sternomastoid and sternohyoid muscles and secured within a silastic covered bipolar platinum/iridium electrode (MicroProbes for Life Science). Charge-balanced biphasic stimulation was delivered at 0.75 mA, 0.20 ms pulse width, 10 Hz for 60 s by a custom stimulator (SetPoint Medical). In some animals, the nerve was stimulated with a single biphasic pulse, 0.1 s, at 1.0 mA. In sham-operated animals the vagus nerve was visualized but not manipulated. Skin was closed with surgical staples and the mice recovered.

### Rodent endotoxemia

At various times post-VNS and sham-VNS (0, 2, 24, 48, and 72 h in rat, 24 h in mouse) VNS, endotoxin (0111:B4; Sigma-Aldrich) was injected intraperitoneally (IP; 1.5 mg/kg in rats; 8 mg/kg in mice). Rodents were euthanized after 90 min and serum TNF concentrations were measured using ELISA (R&D Systems).

### Macrophage cultures

RAW264.7 cells were incubated with 0 or 10 μM acetylcholine in serum-free Opti-Mem media (Gibco) containing 1 mM pyridostigmine bromide (Sigma-Aldrich), for 60 min. Media was collected 4 h after endotoxin (10 ng/ml LPS) exposure and analyzed for TNF by ELISA (R&D Systems).

Human blood was collected from healthy subjects. Peripheral blood mononuclear cells (PBMC) were collected and monocytes isolated using the Monocyte Isolation Kit II (Miltenyi Biotec). Human monocyte and macrophage cultures were exposed to 10 μM acetylcholine chloride (Sigma-Aldrich) in serum-free Opti-Mem media (Gibco) for 60 min in the presence of 1 mM pyridostigmine bromide. Cultures were washed to remove the cholinergic agonist and challenged with endotoxin in serum-free Opti-Mem media (Gibco) 24 h later. Media was collected 4 h after endotoxin addition and analyzed for TNF by ELISA (R&D Systems).

### cAMP and adenylyl cyclase assay

Bone-marrow derived macrophages from wild type (*n* = 2) and α7nAChR^−/−^ (*n* = 3) mice were obtained by flushing the femurs and tibia with sterile PBS. After 6 days of culture in the presence of 20 ng/ml M-CSF (PeproTech), cells were scraped, re-plated, and exposed to the α7nAChR selective agonist PHA568487 (Tocris) for 15 min. Wells were then washed in ice cold PBS, and cell lysis buffer (R&D systems) added on dry ice. Competitive cAMP ELISAs were conducted according to manufacturer instructions (R&D systems). Samples were freeze-thawed twice and centrifuged before assay. Protein concentration was determined by the Bradford assay (Bio-Rad) and used for normalization of cAMP values.

RAW264.7 cells were incubated with the indicated concentrations of [cis-N-(2-Phenylcyclopentyl)azacyclotridec-1-en-2-amine.HCl] (MDL 12,330A; Sigma Aldrich) for 30 min, followed by 50 mM choline chloride for 30 min. Endotoxin was added for 3 h. RNA was then isolated, cDNA generated, and analyzed by qPCR. Data shown (normalized to GAPDH) are representative of three (3) independent experiments and expressed as fold increase relative to unstimulated controls.

### siRNA transfection

siRNA (125 pmol, Dharmacon) directed against adenylyl cyclase 6 (M-043463-01) or c-Fos (L-041157-00), or scrambled siRNA (D-001206-14-20), were transfected into RAW264.7 cells. 72 h later, cells were incubated with 50 mM choline for 30 min, followed by 100 ng/ml LPS for 3 h. RNA levels were analyzed by qPCR. Data shown (normalized to GAPDH) are representative of three independent experiments and expressed as fold increase relative to unstimulated controls.

### Western blot

Protein was extracted from cell lysates and Western blot was performed using the anti-AC5/6 (sc-590, Santa Cruz Biotechnology), anti-c-Fos (sc-52, Santa Cruz Biotechnology), goat-anti-human CREB (AF2989, R&D Systems), rabbit anti-phospho-CREB (AF2510, R&D Systems), and anti-tubulin (05-661, Millipore) antibodies.

### ACREB virus transduction

Human macrophage cultures were transduced (Lipofectamine® 2000, ThermoFisher) with a replication deficient adenovirus (35) encoding green fluorescent protein (GFP) and a phosphorylation defective CREB (ACREB) (32) or a control virus encoding GFP. Three days after exposure to virus, cells were incubated with 100 μM 3-[(3E)-3-[(2,4-dimethoxyphenyl)methylidene]-5,6-dihydro-4H-pyridin-2-yl]pyridine (GTS21), a stable and selective α7nAChR agonist (36, 37), for 2 h, washed, and then cultured for 24 h. Endotoxin was added to the cultures for 4 h, then media collected for measurement of TNF by ELISA (R&D systems).

### Statistical analysis

Differences between experimental groups were evaluated using two-tailed Student's *t*-test or ANOVA followed by Tukey *post-hoc* analysis as appropriate. *p* < 0.05 was considered significant.

## Author contributions

LT: project development, manuscript writing, and manuscript editing. PO and KT: project development, data collection, data analysis, manuscript writing, and manuscript editing. CR, MR-B, MT, AD, LH, WH, JL, WP, KO, YA-A, MF, VP, UA, SC, YL, TM, and KT: project development, data collection, data analysis, and manuscript editing. AC: manuscript writing and manuscript editing.

### Conflict of interest statement

The authors declare that the research was conducted in the absence of any commercial or financial relationships that could be construed as a potential conflict of interest.
